# Health Care Provider Advice for African American Adults Not Meeting Health Behavior Recommendations

**Published:** 2006-03-15

**Authors:** Elizabeth A Fallon, Sara Wilcox, Marilyn Laken

**Affiliations:** Department of Exercise Science, Arnold School of Public Health, University of South Carolina; Arnold School of Public Health, University of South Carolina, Columbia, SC; College of Nursing, Medical University of South Carolina, Charleston, SC

## Abstract

**Introduction:**

Poor dietary habits and sedentary lifestyle contribute to excessive morbidity and mortality. *Healthy People 2010* goals are for 85% of physicians to counsel their patients about physical activity and for 75% of physician office visits made by patients with cardiovascular disease, diabetes, or dyslipidemia to include dietary counseling. The purpose of this study was to 1) determine the rate of participant-reported health care provider advice for healthy lifestyle changes among African Americans who do not meet recommendations for physical activity, fruit and vegetable consumption, and healthy weight; 2) examine correlates of provider advice; and 3) assess the association between provider advice and stage of readiness for change for each of these health behaviors.

**Methods:**

Data for this study were collected as part of a statewide faith-based physical activity program for African Americans. A stratified random sample of 20 African Methodist Episcopal churches in South Carolina was selected to participate in a telephone survey of members aged 18 years and older. The telephone survey, conducted over a 5-month period, asked participants a series of questions about sociodemographics, health status, physical activity, and nutrition. Analyses for moderate to vigorous physical activity, fruit and vegetable consumption, and weight loss were conducted separately. For each of these behaviors, logistic regression analyses were performed to examine the independent association of sex, age, body mass index, education, number of diagnosed diseases, perceived health, and stage of change with health care provider advice for health behaviors.

**Results:**

A total of 572 church members (407 women, 165 men; mean age, 53.9 years; range, 18–102 years) completed the survey. Overall, participant-reported provider advice for lifestyle changes was 47.0% for physical activity, 38.7% for fruit and vegetable consumption, and 39.7% for weight. A greater number of diagnosed diseases and higher body mass index were independently associated with receiving advice to increase physical activity. A more advanced stage of change and a greater number of diagnosed diseases were independently associated with receiving advice for fruit and vegetable consumption. Body mass index, stage of change, and poorer perceived health were independently associated with receiving advice about weight.

**Conclusion:**

Health care provider advice appears to be based predominantly on comorbidities. Because of the preventive benefit of physical activity, fruit and vegetable consumption, and healthy weight, all health care providers are urged to increase counseling for all patients not meeting health behavior recommendations.

## Introduction

In the United States in 2002, 890 million visits were made to physician offices, or approximately 314.4 visits per 100 people (1). Because physician advice is highly valued by patients (2,3), the health care system is an optimal environment for influencing lifestyle changes. *Healthy People 2010*, a nationwide health promotion and disease prevention agenda (4), has set the following goals for advice to patients about health behaviors: 1) 85% of physicians will counsel their patients about physical activity, and 2) 75% of physician office visits made by patients with cardiovascular disease, diabetes, or dyslipidemia will include dietary counseling. These goals are based on evidence that poor dietary habits and a sedentary lifestyle contribute to excessive morbidity and mortality and that physician counseling for diet and physical activity is effective but not practiced often enough to have a significant impact on patient behavior (4). Since the publication of *Healthy People 2010*, research has continued to document low levels of physician counseling for physical activity, fruit and vegetable consumption, and weight control. Reported rates of physician counseling are as low as 28% for physical activity, 40% for nutrition, and 12% for weight loss (5-7).

Although these rates of physician counseling seem low, there are three limitations that may have affected this research. First, researchers examining physician counseling rates for these health behaviors may not have considered whether patients were already meeting guidelines (6-10). For example, if a physician appropriately assessed a patient as already meeting the guidelines for physical activity, fruit and vegetable consumption, and weight, it may not be practical for the physician to spend time counseling the patient on these behaviors. Instead, it is better use of time to counsel patients about behaviors for which they are in greatest need of advice (e.g., stress reduction, alcohol consumption). Additionally, many studies have overlooked the impact of health care professionals other than physicians on patients' lifestyle behaviors (5,8,10-12). During an office visit, a patient spends only about 10 minutes with a physician (13,14), and any additional time is spent with nurses, physician's assistants, or both. Thus, some patients not receiving advice from their physician during an office visit may be receiving it from other health care professionals. Because of these considerations, research on the prevalence of provider advice for health behavior changes should assess advice from all health care providers and include only patients who are not meeting recommendations for the health behavior of interest.

A second limitation of research on health care provider advice is that many studies have focused on predominantly white populations (5,8,10). Examining rates of provider advice for health behaviors and correlates of provider advice in African American populations is important because compared with whites, African Americans are less active, eat fewer servings of fruits and vegetables, and are more likely to be diagnosed with chronic diseases related to inactivity and overweight or obesity (15,16). Furthermore, because the annual rate of office visits for whites (334.6 visits per 100 people) is higher than for African Americans (252.9 visits per 100 people), the importance of health behavior counseling increases for each office visit made by an African American patient (1).

Third, it is important to note that few studies have examined the relationship between provider advice and patients' readiness to change (10). Because behavior change is a process that involves an individual's motivation to change and may require several attempts before long-term success is achieved (17-19)Third, it is important to note that few studies have examined the relationship between provider advice and patients' readiness to change (10). Because behavior change is a process that involves an individual's motivation to change and may require several attempts before long-term success is achieved (17-19), it is important to assess an individual's readiness to change. According to the transtheoretical model, behavior change occurs through periods of progression and relapse as an individual moves through a five-stage process: 1) precontemplation, 2) contemplation, 3) preparation, 4) action, and 5) maintenance (18,19). These five stages of change are based on current behavior, intention to change, and the time frame for behavior or intention.

The goals of this study were to 1) determine the rate of health care provider advice for healthy lifestyle changes in African Americans who do not meet recommendations for physical activity, fruit and vegetable consumption, and weight; 2) examine the correlates of provider advice such as age, education, income, sex, perceived health, and diagnosed diseases (i.e., cardiovascular disease, diabetes, high blood pressure, and high cholesterol); and 3) assess the association between provider advice and stage of change for each of these health behaviors.

## Methods

### Participants

Data for this study were collected as part of a statewide faith-based physical activity program for African Americans in South Carolina (20). The original project was reviewed and approved by the institutional review boards at the Medical University of South Carolina (MUSC) and University of South Carolina (USC) and a planning committee composed of representatives of MUSC, USC, and the African Methodist Episcopal (AME) church. Approximately 25% of South Carolina's African American population is affiliated with the AME church, and there are more than 500 churches in the 7th Episcopal District. A stratified random sample of 20 AME churches in this district was selected to participate, ensuring that churches of various sizes in each of the six geographical areas of the state were selected. The pastors of the selected churches were asked to provide rosters of membership to the USC research staff after giving church members the opportunity to remove their names from the rosters. After duplicate households and telephone numbers were removed, a total nonduplicated sample of 1508 telephone numbers was attempted. A telephone survey of these church members was conducted over a 5-month period by the Institute for Public Service and Policy Research Survey Research Laboratory at USC using computer-aided telephone interviewing (CATI) software (Ci3 System, Sawtooth Technologies, Northbrook, Ill). To be eligible for the survey, participants had to be at least 18 years of age. Because the data reported in this paper are from the baseline sample of an intervention study within AME churches, participants also had to attend AME church services at least twice per month to ensure exposure to the intervention. The survey consisted of questions about sociodemographics, health, physical activity, and nutrition and lasted 20 to 30 minutes.

A total of 572 church members completed the survey; 501 were ineligible, and 167 refused. For response and refusal rates, the denominator equaled the number of names randomly selected from the rosters by the Survey Research Laboratory minus the number of respondents who were reached but determined to be ineligible for the study. The overall response rate was 56.8%. The overall refusal rate was 16.6%. No compensation was provided for participation. Only measures used in the present study are described.

### Measures

#### Sociodemographics

Age, sex, education, and income were assessed using questions from the Centers for Disease Control and Prevention's (CDC's) 2001 Behavioral Risk Factor Surveillance System (BRFSS) (21). Age was a continuous variable. Three categories were created for education (less than or equal to a high school degree, some college, and college graduate), and three were created for annual income (less than $20,000, $20,000 to $35,000, and greater than $35,000).

#### Body mass index

Self-reported height and weight measurements were used to calculate body mass index (BMI) (weight [kg]/height [m^2^]). Participants with a BMI <25 kg/m^2^ were classified as normal weight, participants with a BMI ≥25 kg/m^2^ but <30 kg/m^2^ were classified as overweight, and participants with a BMI ≥30 kg/m^2^ were classified as obese. Participants with a BMI ≥25 kg/m^2^ were classified as not meeting recommendations for healthy weight.

#### Perceived health

Participants rated their health on a 5-point Likert scale from the BRFSS (22) with the following response options: excellent = 5; very good = 4; good = 3; fair = 2; and poor = 1.

#### Diagnosed diseases

Participants were asked four questions from the BRFSS (22) about whether they had been diagnosed with chronic diseases related to physical activity and dietary habits. Each question began, "Has a doctor, nurse, or other health professional ever told you that you had" and ended with 1) "high blood pressure," 2) "high cholesterol," 3) "diabetes," and 4) "cardiovascular disease." Each yes response was counted as a diagnosed health condition; the total number of yes responses was added for each participant (range 0 to 4). Three categories were created for analysis on the basis of score frequency: 0, 1, and 2 or more diagnosed diseases.

#### Physical activity

Physical activity levels were assessed using the BRFSS physical activity module (22). Examples of moderate and vigorous physical activity were provided. The frequency and duration of activity were assessed for moderate and vigorous physical activity. Recently, a study comparing an objective physical activity monitoring technique with the BRFSS physical activity module reported 80% agreement between the two methods of classifying individuals who met current CDC and American College of Sports Medicine (ACSM) recommendations for physical activity (23). Participants were classified as meeting recommendations for moderate to vigorous physical activity if they participated in moderate physical activity at least 5 days per week for at least 30 minutes at a time or participated in vigorous physical activity at least 3 days per week for at least 20 minutes at a time.

#### Fruit and vegetable consumption

Fruit and vegetable consumption was assessed by asking questions adapted from the BRFSS (22). Participants were asked, "How many servings of fruit do you usually eat each day?" and "How many servings of vegetables do you usually eat each day?" Responses were summed to determine whether participants were meeting current recommendations (i.e., at least five servings of fruits and vegetables per day).

#### Readiness for change

The stage of change for moderate to vigorous physical activity and fruit and vegetable consumption was assessed using a three-question staging algorithm from the Behavior Change Consortium (24). Because of low frequency counts in some stages for moderate to vigorous physical activity and fruit and vegetable consumption, participants were grouped into either precontemplation (stage 1) or contemplation/preparation (stages 2 or 3). Because the focus was on individuals not meeting recommendations, no participants were in stages 4 or 5. For weight loss, participants were categorized into either a pre-action or an action stage on the basis of their response to the question, "Are you currently trying to lose weight?"

#### Health care provider counseling

Each participant was asked about provider advice for each health behavior of interest. Participants were asked, "In the past 12 months, has a doctor, nurse, or other health professional told you to be more physically active?," "In the past 12 months, has a doctor, nurse, or other health professional told you to eat more fruits and vegetables?," and "In the past 12 months, has a doctor, nurse or other health professional given you advice about your weight?"

### Statistical analyses

Analyses for moderate to vigorous physical activity, fruit and vegetable consumption, and weight loss were conducted separately. For each analysis, survey participants who were not meeting recommendations were selected. Thus, the number of participants included in each analysis varied. For each of the three behaviors, logistic regression analyses were used to examine the independent association of sex, age, BMI, education, number of diagnosed diseases, perceived health, and stage of change with provider advice for health behaviors. Income was not included in the univariate and multivariate analyses because of its strong association with education. Whether or not the participant received provider advice for the health behavior of interest was the dichotomous dependent variable. For each independent variable, the referent group was the group hypothesized to be least likely to receive provider advice. Thus, each odds ratio can be interpreted as indicating a greater chance of receiving provider advice relative to the referent group. Finally, to determine which factors were independently associated with provider advice for moderate to vigorous physical activity, fruit and vegetable consumption, and weight, the continuous variables (BMI, age, and perceived health) and categorical variables (sex, education, diagnosed diseases, and stage of change) were forced into the model simultaneously.

## Results

### Survey participants

The survey sample included 572 participants (407 women and 165 men; mean age, 53.9 years; age range, 18–102 years). Descriptive statistics for participants not meeting recommendations for each of the three health categories are shown in Table 1, Table 2, and Table 3. Among the 165 men in the sample, 11 (6.7%) did not report their weight, 43 (26.1%) were classified as having a healthy weight, 60 (36.4%) were classified as overweight, and 51 (30.9%) were classified as obese; 55 (33.5%) reported having been diagnosed with one chronic disease, and another 55 (33.5%) reported having been diagnosed with two or more chronic diseases. For the univariate and multivariate analyses, the sample sizes for the men not meeting recommendations were 103 for moderate to vigorous physical activity, 131 for fruit and vegetable consumption, and 111 for overweight.

Among the 407 women in the sample, 44 (10.8%) did not report their weight, 86 (21.1%) were classified as having a healthy weight, 125 (30.7%) were classified as overweight, and 152 (37.3%) were classified as obese; 140 (34.4%) of the women reported having been diagnosed with one chronic disease, and another 131 (32.2%) reported having been diagnosed with two or more chronic diseases. For the univariate and multivariate analyses, the sample sizes for the women not meeting recommendations were 282 for moderate to vigorous physical activity, 262 for fruit and vegetable consumption, and 277 for overweight.

### Moderate to vigorous physical activity

In univariate analyses (Table 4), the likelihood of provider counseling for moderate to vigorous physical activity increased as age (*P* = .02) and BMI (*P* < .001) increased. The poorer participants perceived their health to be, the more likely they were to receive advice to increase moderate to vigorous physical activity (*P* < .001). Compared with participants with no diagnosed diseases, participants with one diagnosed disease were more than two and a half times as likely to receive advice to increase moderate to vigorous physical activity (*P* < .001), and those with two or more diagnosed diseases were four times as likely to receive advice to increase moderate to vigorous physical activity (*P* < .001). The association between sex and advice (*P* = .09) approached significance such that women were more likely to be advised than men. Similarly, the association between stage of change and advice (*P* = .08) approached significance such that those who received advice were more likely to be in a higher stage of change for moderate to vigorous physical activity. Finally, advice to increase moderate to vigorous physical activity was not associated with education.

In the multivariate analysis (Table 5), two factors were independently associated with advice to increase moderate to vigorous physical activity (model χ^2^_9_ [N = 329] = 50.25, *P* < .001). Number of diagnosed diseases was the strongest correlate of provider advice (*P* < .001), followed by BMI (*P* = .009). Thus, having been diagnosed with chronic diseases (Wald χ^2^ = 18.07, *P* < .001) and having a higher BMI (Wald χ^2^ = 6.90, *P* = .009) were associated with receiving advice from health care providers to increase moderate to vigorous physical activity. The association between stage of change and advice (*P* = .07) approached significance such that those who received advice were more likely to be in a higher stage of change for physical activity.

### Fruit and vegetable consumption

In univariate analyses (Table 4), participants with a higher BMI (*P* = .05) and poorer perceived health (*P* = .002) were more likely to be counseled for fruit and vegetable consumption. Participants with one diagnosed disease were more than two times as likely to receive advice to increase fruit and vegetable consumption (*P* = .003) as participants with no diagnosed diseases, and participants with two or more diagnosed diseases were almost three times as likely to receive advice to increase fruit and vegetable consumption (*P*  < .001). Participants in the contemplation or preparation stages were more than two times as likely to be advised to increase fruit and vegetable consumption (*P* < .001) as participants in the precontemplation stage. The association between sex and advice (*P* = .06) and the association between education and advice (*P* = .09) approached significance, such that women and those with more than a high school education were more likely to receive advice to increase fruit and vegetable consumption.

When all independent variables were forced into the multivariate model (Table 5), two factors were independently associated with whether a participant had received advice to increase fruit and vegetable consumption (model χ^2^_9_ [N = 338] = 32.74, *P* < .001). Stage of change was the strongest correlate of advice (*P* = .001), followed by number of diagnosed diseases (*P* = .01). The model correctly classified 65.7% of the cases. Thus, being in a higher stage of change (Wald χ^2^ = 11.12, *P* = .001) and having been diagnosed with chronic diseases (Wald χ^2^ = 9.00, *P* = .01) were associated with receiving advice from health care providers to increase fruit and vegetable consumption.

### Weight loss

Univariate analyses (Table 4) indicate that women were almost two times as likely as men to receive advice about their weight (*P* = .006). Higher BMI (*P* < .001) and poorer perceived health (*P* = .008) were associated with advice about weight. Likewise, participants with one (*P* = .02) or two or more (*P* = .009) diagnosed diseases were about two times as likely to receive advice about their weight. Participants currently trying to lose weight (i.e., action stage) were more than two times as likely to receive advice about their weight as participants not currently trying to lose weight (i.e., pre-action stage, *P* < .001).

When all variables were forced into the multivariate model (Table 5), three factors were statistically reliable in distinguishing whether participants had received advice about weight (model χ^2^_9 _[N = 371] = 51.18, *P* < .001). Wald statistics indicated that BMI was the strongest correlate of advice (Wald χ^2^ = 14.29, *P* < .001), followed by stage of change (Wald χ^2^ = 5.94, *P* = .02) and perceived health (Wald χ^2^ = 3.85, *P* = .05). The model correctly classified 64.7% of the cases. Thus, participants with a higher BMI, participants currently trying to lose weight, and participants reporting poorer perceived health were most likely to receive advice from health care providers about weight.

## Discussion

Overall, participant-reported health care provider advice for healthy lifestyle changes was fairly low in African Americans not meeting recommendations. Of the participants, 47.0% were counseled for moderate to vigorous physical activity, 38.7% were counseled for fruit and vegetable consumption, and 39.7% were counseled for weight. Although the rates of health care provider advice seem to be higher than in previous reports (5-7), they still have not reached goals set by *Healthy People 2010* (4). In multivariate analyses, results indicated that a higher number of diagnosed diseases and greater BMI were independently associated with receiving advice for physical activity. For fruit and vegetable consumption, a more advanced stage of change and greater number of diagnosed diseases were independently associated with receiving advice. Finally, weight, BMI, stage of change, and perceived health were independently associated with receiving health care provider advice.

The key finding of our study is that provider advice to increase healthy lifestyle behaviors was associated with the presence of patient comorbidities. Specifically, the presence of chronic diseases was the major criterion for provider advice for physical activity and fruit and vegetable consumption; participants diagnosed with at least one chronic disease were at least two times as likely to receive advice for moderate to vigorous physical activity and fruit and vegetable consumption as those with no diagnosed chronic diseases. Thus, instead of inquiring about the health behaviors themselves, it appears that health care providers are using the presence of comorbidities to guide their counseling for healthy lifestyle changes.

This interpretation that health care providers are counseling on the basis of comorbities is supported by the result that participants with a higher BMI were more likely to receive advice about weight and physical activity. Although overweight and obesity are not considered diseases, they are major risk factors for disease, and instead of counseling patients about their weight and physical activity as a preventive approach, it appears that physicians wait until a patient is overweight before counseling for physical activity and weight. BMI was not independently associated with advice for fruit and vegetable consumption in multivariate analyses. This is an important finding because healthy weight loss is most successful when simultaneously increasing physical activity and reducing caloric consumption (which is often achieved through diets promoting increases in fruits and vegetables [24,25]). Thus, our results corroborate recent evidence that many patients, providers, or both may not be making the connection between the need to lose weight and the need to simultaneously increase physical activity and eat diets high in fruit and vegetables (26).

Another important finding in this study is that, similar to other studies (6,27), a greater percentage of women reported being counseled for physical activity, fruit and vegetable consumption, and weight. Although this difference was statistically significant only for weight, these data suggest that men are less likely to receive advice from their health care providers to change lifestyle behaviors. It is possible that providers are more likely to give advice to women because they feel more comfortable counseling women or because they feel as though their advice is more effective for women than men. Another possibility is that women may be more likely to receive advice because they visit health care providers more often than men do because of gynecological and pediatric visits; women may also spend more minutes per visit with the physician (28). Alternatively, one can make the argument that the patient's sex does not moderate provider counseling but that women are more likely than men to remember being counseled or are more likely to follow provider advice (29). Future researchers can resolve these issues by investigating sex as a moderating variable for health care provider counseling as well as recording interactions between health care providers and patients rather than relying on self-reported information.

Consistent with previous research (5,6,8,12,27), older participants were significantly more likely to receive advice to increase moderate to vigorous physical activity. This association, however, was not present for fruit and vegetable consumption or weight. Furthermore, in multivariate analyses, age did not retain its independent effect on advice for physical activity. The equivocal nature of the relationship between age and provider advice may indicate the presence of a mediating variable. Providers may not specifically use age as a criterion for giving advice for behavior change but instead may use incidence of chronic disease (which increases with age).

In contrast to previous research (8,27), provider advice was not associated with education level for any of the health behaviors investigated in this study. It is important to note, however, that our sample was substantially smaller than other studies (8,27); the smaller sample size may have limited our ability to detect the effect of education on health behavior. Because of the equivocal results related to education, it is possible that the association between advice and education is mediated by the quality and frequency of health care and not directly due to the use of education level as a variable to determine whether patients are advised for health behaviors.

Finally, we found that health care provider advice was associated with readiness for change for weight loss and fruit and vegetable consumption (10). Because of the cross-sectional nature of the data, this result could be interpreted in two ways. First, it might be argued that patients moved into a higher stage of change after receiving advice from their health care provider to change behavior. It could also be argued, however, that participants in a higher stage of change may have been more likely to seek advice from their provider or that those in a higher stage of change were more likely to remember and adhere to their provider's advice. Recent research has suggested, however, that participants in a higher stage of change are more likely to seek provider advice; provider advice for lifestyle change has been shown to be an effective tool in changing physical activity and dietary behaviors (29-32).

A limitation of this study is its reliance on cross-sectional data; no cause–effect relationship between provider advice and its correlates can be established. Additionally, it is important to note the limitations of self-reported data (33). Participants may forget, misunderstand, or otherwise unintentionally misreport retrospective information such as current levels of physical activity, fruit and vegetable consumption, weight, and the exact nature of the physician's advice. Therefore, research investigating the difference between actual and perceived counseling is needed. However, even if reality and perceptions do not match, it remains important to understand patients' perceptions of health care provider advice, as perceptions are likely to be most influential in affecting a patient's decision to change. Another limitation to this study is that we did not ask participants whether they were counseled for dietary behaviors other than fruit and vegetable consumption. It is possible that participants were told by their health care providers to reduce dietary fat, reduce sugar, or increase fiber. Thus, future research may ask participants whether their health care providers told them to change their eating habits and then follow up by asking them what changes the provider asked them to make.

This study has improved upon this area of research by 1) focusing on individuals who did not meet current recommendations for the behavior of interest, 2) surveying a large African American population, and 3) assessing the relationship between health care provider advice and patients' readiness for change. We found that the rates of health care provider advice for moderate to vigorous physical activity, fruit and vegetable consumption, and weight continue to fall short of the goals set by *Healthy People 2010*. We also found that provider advice is not based on patients' behaviors but on comorbidities associated with the absence of these behaviors, thus substantiating previous research indicating that individuals who are the least healthy (e.g., high BMI, diagnosed with chronic health conditions) are most likely to be advised by health care providers to change their behaviors. Furthermore, our results indicate that health care provider advice is associated with readiness to change, and it is likely that this (predisease) advice would have been taken into account by patients. Thus, because of the preventive benefit of physical activity, fruit and vegetable consumption, and healthy weight, health care providers are urged to increase counseling for all patients (i.e., healthy and chronically ill). Further research is warranted for the overall effect of provider counseling as well as the identification of moderators (i.e., sex) and mediators for the effect of provider counseling on behavior change.

## Figures and Tables

**Table 1:** Characteristics of Survey Participants (n = 385) Not Meeting Recommendations for Moderate to Vigorous Physical Activity

**Characteristic**	**Received Advice From Provider**	**Did Not Receive Advice From Provider**

**Continuous variables**	**Mean (SD)**	**Mean (SD)**
Body mass index (range, 17.1–54.6)	30.35 (6.37)	27.97 (5.33)
Age, y (range, 18–102)	56.4 (14.1)	52.6 (16.2)
Perceived health^a^	3.0 (1.0)	3.3 (1.0)

**Categorical variables**	**No. (%)**	**No. (%)**

**Sex **		

Male	41 (39.8)	62 (60.2)
Female	140 (49.6)	142 (50.6)

**Education**		

≤High school degree	98 (50.8)	95 (49.2)
Some college	37 (43.0)	49 (57.0)
≥College graduate	42 (45.2)	51 (54.8)

**Annual income, $**		

<20,000	49 (52.1)	45 (47.9)
20,000–35,000	50 (43.9)	64 (56.1)
>35,000	66 (48.9)	69 (51.1)

**Diagnosed diseases**		

None	34 (27.9)	88 (72.1)
1 Disease	66 (50.8)	64 (49.2)
≥2 Diseases	80 (61.1)	51 (38.9)

**Stage of change**		

Precontemplation	46 (40.0)	69 (60.0)
Contemplation or preparation	128 (49.8)	129 (50.2)

a Participants rated their health on a 5-point Likert scale, with 1 = poor, 2 = fair, 3 = good, 4 = very good, and 5 = excellent.

**Table 2:** Characteristics of Survey Participants Not Meeting Recommendations for Fruit and Vegetable Consumption (n = 393)

**Characteristic**	**Received Advice From Provider**	**Did Not Receive Advice From Provider**

**Continuous variables**	**Mean (SD)**	**Mean (SD)**
Body mass index (range, 15.9–54.6)	29.65 (6.51)	28.42 (5.57)
Age, y (range, 18–102)	54.6 (14.4)	52.9 (15.8)
Perceived health^a^	3.1 (1.0)	3.4 (1.1)

**Categorical variables**	**No. (%)**	**No. (%)**

**Sex **		

Male	42 (32.1)	89 (67.9)
Female	110 (42.0)	152 (58.0)

**Education**		

≤High school degree	93 (44.3)	117 (55.7)
Some college	28 (33.3)	56 (66.7)
≥College graduate	31 (33.0)	63 (67.0)

**Annual income, $**		

<20,000	50 (52.6)	45 (47.4)
20,000–35,000	44 (34.6)	83 (65.4)
>35,000	50 (36.0)	89 (64.0)

**Diagnosed diseases**		

None	33 (25.0)	99 (75.0)
1 Disease	58 (42.0)	80 (58.0)
≥2 Diseases	60 (49.2)	62 (50.8)

**Stage of change**		

Precontemplation	44 (27.8)	114 (72.2)
Contemplation or preparation	99 (47.6)	109 (52.4)

a Participants rated their health on a 5-point Likert scale, with 1 = poor, 2 = fair, 3 = good, 4 = very good, and 5 = excellent.

**Table 3:** Characteristics of Survey Participants Not Meeting Recommendations for Weight (n = 388)

**Characteristic**	**Received Advice From Provider**	**Did Not Receive Advice From Provider**

**Continuous variables**	**Mean (SD)**	**Mean (SD)**
Body mass index (range, 25.0–54.6)	32.87 (5.64)	30.20 (4.13)
Age, y (range, 19–102)	53.3 (14.6)	53.6 (14.6)
Perceived health^a^	3.1 (1.0)	3.3 (1.0)

**Categorical variables**	**No. (%)**	**No. (%)**

**Sex **		

Male	32 (28.8)	79 (71.2)
Female	122 (44.0)	155 (56.0)

**Education**		

≤High school degree	79 (40.1)	118 (59.9)
Some college	31 (36.0)	55 (64.0)
≥College graduate	44 (43.6)	57 (56.4)

**Annual income, $**		

<20,000	38 (40.4)	56 (59.6)
20,000–35,000	47 (38.2)	76 (61.8)
>35,000	57 (39.3)	88 (60.7)

**Diagnosed diseases**		

No diseases	32 (28.8)	79 (71.2)
1 Disease	61 (43.6)	79 (56.4)
≥2 Diseases	61 (45.2)	74 (54.8)

**Stage of change**		

Preaction	43 (27.6)	113 (72.4)
Action	111 (47.8)	121 (52.2)

a Participants rated their health on a 5-point Likert scale, with 1 = poor, 2 = fair, 3 = good, 4 = very good, and 5 = excellent.

**Table 4:** Likelihood of Receiving Health Care Provider Advice for Health Behaviors, by Individual Correlates

** Correlate**	**Received Provider Advice for Behavior**					

	**Moderate to Vigorous Physical Activity(n = 385)**		**Fruit and Vegetable Consumption(n = 393)**		**Weight(n = 388)**	

	**OR (95% CI)^a^**	***P* Value**	**OR (95% CI)^a^**	***P* Value**	**OR (95% CI)^a^**	***P* Value**
**Sex**						
Male	1.00		1.00		1.00	
Female	1.49 (0.94-2.36)	.09	1.53 (0.99-2.39)	.06	1.94 (1.21-3.12)	.006
**Age^b^**	1.02 (1.003-1.03)	.02	1.01 (0.99-1.02)	.29	1.00 (0.98-1.01)	.82
**Body mass index^c^**	1.07 (1.03-1.11)	<.001	1.04 (1.001-1.07)	.05	1.12 (1.07-1.17)	<.001
**Education**						
≤High school degree	1.00		1.00		1.00	
Some college	0.73 (0.44-1.22)	.23	0.63 (0.37-1.07)	.09	0.84 (0.50-1.42)	.52
≥College graduate	0.80 (0.49-1.31)	.37	0.62 (0.37-1.03)	.07	1.15 (0.71-1.87)	.57
**Perceived health^d^**	0.69 (0.56-0.85)	<.001	0.73 (0.60-0.90)	.002	0.76 (0.62-0.93)	.008
**Diagnosed diseases**						
No diseases	1.00		1.00		1.00	
1 Disease	2.67 (1.58-4.51)	<.001	2.18 (1.29-3.66)	.003	1.91 (1.12-3.24)	.02
≥2 Diseases	4.06 (2.32-6.89)	<.001	2.90 (1.71-4.93)	<.001	2.04 (1.20-3.47)	.009
**Stage of change**						
Precontemplation	1.00		1.00		NA^e^	NA
Contemplation or preparation	1.49 (0.95-2.33)	.08	2.35 (1.51-3.66)	<.001	NA	NA
Preaction	NA	NA	NA	NA	1.00	
Action	NA	NA	NA	NA	2.41 (1.56-3.73)	<.001

a OR indicates odds ratio; CI, confidence interval.

b ORs for age indicate increased likelihood of receiving advice for the behavior with one additional year of age.

c ORs for body mass index indicate increased likelihood of receiving advice for the behavior with an increase in body mass index of 1.0 kg/m^2^.

d ORs for perceived health indicate increased likelihood of receiving advice with a decrease of 1 point on the perceived health scale. Perceived health was rated by participants on a 5-point Likert scale, with 1 = poor, 2 = fair, 3 = good, 4 = very good, and 5 = excellent.

e NA indicates not applicable.

**Table 5:** Significant Correlates of Receiving Health Care Provider Advice for Health Behaviors in Multivariate Logistic Regressions

** Correlate**	**OR (95% CI)^a^**	**Wald χ^2^_*df* _(*P* Value)**

**Advice on moderate to vigorous physical activity **		

Diagnosed diseases		χ^2^_2_ = 18.07 (<.001)
None	1.00	
1 Disease	2.89 (1.57-2.18)	χ^2^_1 _= 11.56 (.001)
≥2 Diseases	4.53 (2.18-9.43)	χ^2^_1_ = 16.30 (<.001)
Body mass index	1.06 (1.01-1.10)	χ^2^_1_ = 6.90 (.009)

**Advice on fruit and vegetable consumption**		

Stage of change	2.25 (1.40-3.62)	χ^2^_1_ = 11.12 (.001)
Diagnosed diseases		χ^2^_2 _= 9.00 (.01)
None	1.00	
1 disease	2.08 (1.12-3.85)	χ^2^_1 _= 5.43 (.02)
≥2 diseases	3.00 (1.44-6.25)	χ^2^_1_ = 8.59 (.003)

**Advice on weight**		

Body mass index	1.10 (1.05-1.16)	χ^2^_1_ = 14.29 (<.001)
Stage of change	1.86 (1.13-3.06)	χ^2^_1_ = 5.94 (.02)
Perceived health	0.77 (0.60-1.00)	χ^2^_1_ = 3.85 (.05)

a OR indicates odds ratio; CI, confidence interval.
